# Editorial: Diets, Public Health and Environmental Degradation: Trade-Offs, Synergies, and Policy Interventions

**DOI:** 10.3389/fnut.2021.821063

**Published:** 2022-01-17

**Authors:** Tong Wu, Lei Liu

**Affiliations:** ^1^Department of Applied Economics, University of Minnesota Twin Cities, St. Paul, MN, United States; ^2^School of Public Administration, Sichuan University, Chengdu, China

**Keywords:** food security, environmental degradation, food safety, agricultural production, sustainable diets

Global trends in economic development have largely raised the quantity of food supply. However, at the same time, fast economic growth, and urbanization have posed a greater threat to food quality due to significant environmental degradation and shifted diet structure with higher caloric intake and greater consumption of meat and processed foods. These trends and their impacts have had major public health implications. The intensified agricultural production needed to meet this demand, along with the networks of transport used to deliver them, cause further environmental impacts. At the same time, such diets, which have long been prevalent in advanced economies and are increasingly accessible in emerging markets, cause new population-level disease burdens such as obesity/overweight, heart disease, and diabetes.

There is a growing body of interdisciplinary research at this diet-environment-health nexus. Indeed, some scholars have characterized the structural tensions and mutual influences in this triangular relationship as a “trilemma,” in which progress on one or two fronts tends to elicit difficulties on the remaining front(s) (1). In what places, to what extent, and due to what factors these interconnections exist is a new and profitable field of empirical exploration, with major implications for sustainable development (2). Indeed, the COVID-19 pandemic that began sweeping across the world in 2020 has thrown these interconnections into even starker relief, highlighting the urgency of designing and implementing policies that align health and environmental concerns.

Such policies are likely to involve interventions, both *ad-hoc* and structural, to food systems across multiple scales (3, 4). Indeed, it is difficult to imagine how diet-environment-health “trilemmas” and related issues can be resolved without addressing the intensification and expansion of agriculture (especially livestock husbandry), pollution, and waste from food production and processing, as well as food safety concerns related to ineffective monitoring and regulation. These challenges are arguably most pressing in developing countries, where large-scale dietary changes (including convergence with Western diets) are occurring alongside—and in many ways connected to—acute environmental problems and public health risks: the new middle classes of Asia, Africa, and Latin America are often consuming their industrially-produced food amidst heavy air pollution, and in dense urban settings that paved over once-biodiverse habitats.

For the past generation, China has been the world's largest developing country and fastest-growing major economy. As a result, it has arguably also been the world's largest “laboratory” for the myriad social-ecological changes that have necessitated sustainable development (5–7). The preponderance of papers focused on China in this special topic reflects these facts. In an experimental study, Liu et al. analyze the environmental and food safety risks caused by heavy metals from the application of biogas slurry during peanut cultivation. Zou and Zou overview the ecological conditions, distribution, and impacts of pepper production and consumption in China, where the vegetable is a dietary staple. Liu et al. examine how climate change could impact a range of agricultural practices in Northwest China—an agriculturally important but economically laggard region—and suggest policies to promote more sustainable development.

In their country-level analysis, Xian et al. turn their attention to how rural food production and consumption in China affect nitrogen cycling, a critical biogeochemical process greatly influenced by fertilizer use and dietary patterns, and which has major implications for climate change mitigation. Also taking a national perspective, Li et al. identify key trends and policies at the intersection of food safety and pollution, the sources of which have become increasingly diverse and the health impacts of which have become increasingly widespread over the past fifty years. Cong et al. provide an insightful perspective on how food delivery platforms—which are now extensively used—relate to and mitigate obesity/overweight, a public health problem that has worsened alongside rising affluence and urbanization in China. Finally, Hwalla et al. provide an informative overview of problems, prospects, and policy suggestions at the diet-environment-health nexus in Lebanon, a country presently facing many obstacles to sustainable development.

As the papers in this special topic show, identifying tradeoffs and synergies between diet, health, and the environment, along with how these interconnections vary across social-ecological contexts, is a critical task for researchers, policymakers, and stakeholders. While the geographic locus of nearly all the studies is China, they nonetheless cover a diverse set of dietary items, health implications, environmental conditions and impacts, and policy concerns. Additionally, China not only has an outsized role within the global economic system; it is also a key driver of planetary changes and a potential role model—or warning—to other developing countries. Understanding dynamics at the diet-environment-health nexus in China, as well as in a much smaller but also representative (and relatively understudied) nation such as Lebanon, helps advance sustainable development not only within these countries, but also for the world.

## Author Contributions

TW and LL conceived of the ideas and opinions expressed here and jointly wrote the manuscript. All authors contributed to the article and approved the submitted version.

## Conflict of Interest

The authors declare that the research was conducted in the absence of any commercial or financial relationships that could be construed as a potential conflict of interest.

## Publisher's Note

All claims expressed in this article are solely those of the authors and do not necessarily represent those of their affiliated organizations, or those of the publisher, the editors and the reviewers. Any product that may be evaluated in this article, or claim that may be made by its manufacturer, is not guaranteed or endorsed by the publisher.
